# Adult and Developing Zebrafish as Suitable Models for Cardiac Electrophysiology and Pathology in Research and Industry

**DOI:** 10.3389/fphys.2020.607860

**Published:** 2021-01-13

**Authors:** Leyre Echeazarra, Maria Pura Hortigón-Vinagre, Oscar Casis, Mónica Gallego

**Affiliations:** ^1^Departamento de Fisiología, Facultad de Farmacia, Universidad del País Vasco UPV/EHU, Vitoria-Gasteiz, Spain; ^2^Departamento de Bioquímica y Biología Molecular y Genética>, Facultad de Ciencias, Universidad de Extremadura, Badajoz, Spain

**Keywords:** orthologs, arrhythmia, ECG, patch clamp, pharmaceutical, action potential

## Abstract

The electrophysiological behavior of the zebrafish heart is very similar to that of the human heart. In fact, most of the genes that codify the channels and regulatory proteins required for human cardiac function have their orthologs in the zebrafish. The high fecundity, small size, and easy handling make the zebrafish embryos/larvae an interesting candidate to perform whole animal experiments within a plate, offering a reliable and low-cost alternative to replace rodents and larger mammals for the study of cardiac physiology and pathology. The employment of zebrafish embryos/larvae has widened from basic science to industry, being of particular interest for pharmacology studies, since the zebrafish embryo/larva is able to recapitulate a complete and integrated view of cardiac physiology, missed in cell culture. As in the human heart, I_*Kr*_ is the dominant repolarizing current and it is functional as early as 48 h post fertilization. Finally, genome editing techniques such as CRISPR/Cas9 facilitate the humanization of zebrafish embryos/larvae. These techniques allow one to replace zebrafish genes by their human orthologs, making humanized zebrafish embryos/larvae the most promising *in vitro* model, since it allows the recreation of human-organ-like environment, which is especially necessary in cardiac studies due to the implication of dynamic factors, electrical communication, and the paracrine signals in cardiac function.

## Introduction

Mouse is often the first-choice animal model for the study of normal and pathological heart function. However, at the electrophysiological level, the heart of rodents and humans has important functional differences, such as the fact that basal heart rate is more than eight times faster in mice. The murine cardiac action potential (AP) is very short and lacks the characteristic plateau phase of the human AP. Importantly, cardiac repolarization in humans depends mainly on the hERG potassium channel, which is almost absent in the murine heart (Curran et al., 1995; Sanguinetti et al., 1995; Trudeau et al., 1995; Xu et al., 1999; Rosen, 2000; Carmeliet, 2004). These limitations have made necessary the search for alternative or complementary model organisms that resemble better some aspects of the human cardiac physiology and pathology.

The small size of the zebrafish (*Danio rerio*) (adults reach a length of 2–3 cm), easy manipulation, efficient housing, and fast reproduction make it a suitable model for large-scale screenings for research and industry purposes. Another advantage is the possibility they offer to carry out efficient gene targeting methods and high-resolution real-time imaging methods in live fish. It also makes possible to perform large-scale screens of an entire organism, including toxicological, teratogenic, and ecotoxicity studies (Ellis et al., 2014; Zakaria et al., 2018; Cassar et al., 2020). Orthologs for about 70% of the human genes have been found in zebrafish (Howe et al., 2013). The humanization of the zebrafish, by replacing endogenous genes with their human orthologs, enables the recapitulation of several human pathologies, as well as the study of drug toxicity and efficacy in a more precise way (Cornet et al., 2018).

Due to the advantages offered by this experimental model, the zebrafish, initially used for studying the genetics of development and organogenesis, has become a very useful model to study a variety of human pathologies including cancer (Terriente and Pujades, 2013), metabolic diseases such as diabetes or obesity (Zang et al., 2018), neurological and neuropsychiatric diseases (Ijaz and Hoffman, 2016), cardiac pathologies, cardiotoxicity, and drug discovery (Gut et al., 2017; Cassar et al., 2020). The cardiac pathologies include those that affect both the structure and the mechanical functioning of the heart (cardiomyopathies, heart failure (HF), myocardial injury and regeneration, or structural and congenital heart diseases) as well as those that affect the electrical functioning and, therefore, can cause arrhythmias (long QT syndrome, short QT syndrome, Brugada syndrome, sick sinus syndrome, or atrial fibrillation).

Although the zebrafish has a two-chambered heart, with one atrium and one ventricle, the basic electrical properties are very similar to those of the human heart. The cardiac impulse, generated in the sinoatrial (SA) node, pauses at the atrioventricular (AV) junction and then is conducted from apex to base before spreading through the ventricle (Sedmera et al., 2003; Chi et al., 2008). Finally, the morphology and behavior of the zebrafish cardiac AP is similar to that of humans, with a long plateau phase and a clear rate dependence (Nemtsas et al., 2010).

Thanks to the high fecundity of the zebrafish (can lay 50–500 eggs per mating), embryos are readily available (Gut et al., 2017). The heart of zebrafish embryos starts beating approximately at 24 h post fertilization (hpf) and the blood circulates through major vessels by 36 hpf (Stainier et al., 1996). The transparency of zebrafish embryos/larvae makes it possible to observe the developing internal organs (including the heart) under the microscope (see Figure 1 from Crowcombe et al., 2016). On the other hand, mutants with abnormal phenotypes in heart beating and circulation defects led to the identification of genes that are crucial for cardiac morphogenesis and cardiovascular function (Chen et al., 1996; Stainier et al., 1996). In addition, the characterization of ionic channels responsible for the cardiac AP in the early zebrafish embryos/larvae (Alday et al., 2014) further supported its use for studying human cardiac diseases.

**FIGURE 1:**
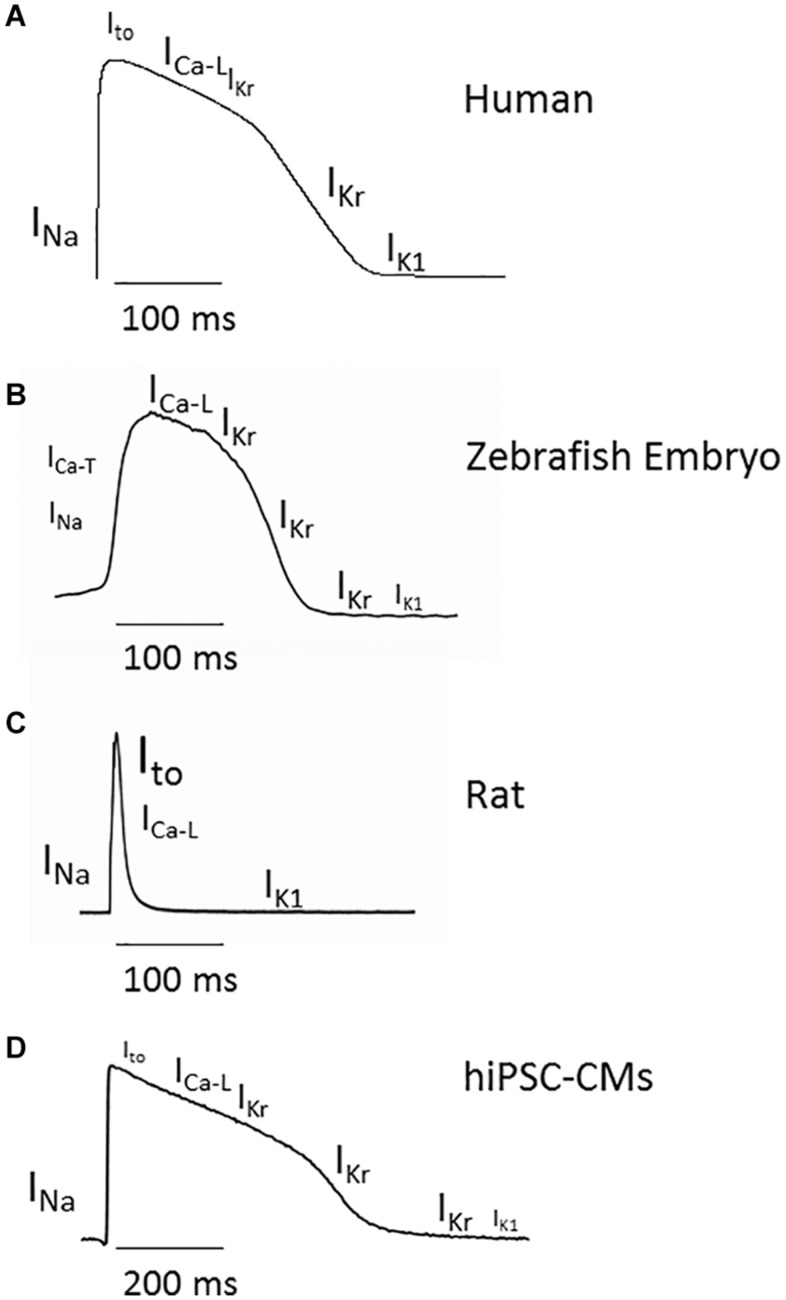
Cardiac action potential (AP) in adult human, zebrafish embryo, rat, and hiPSC-CMs and main ionic currents involved in each phase. **(A)** Adult human ventricular AP profile simulated with the O’Hara-Rudy-dynamic model following the methodology by O’Hara et al. (2011), Passini et al. (2017). **(B)** AP from 48 hpf zebrafish embryo recorded by patch clamp. **(C)** AP from a single rat cardiac ventricular myocyte recorded by patch clamp. **(D)** AP from 2D cultured iCell2 hiPSC-CMs (Cellular Dynamics International, Madison, WI, United States) recorded by CellOPTIQ platform (Clyde Biosciences Ltd., Glasgow, United Kingdom) using voltage-sensitive dyes di4-ANEPPS. Different font sizes represent different current amplitudes.

The increasing use of the zebrafish embryo/larva not only in basic but also in applied research can be explained by the need for biomedical and pharmaceutical industries to have simple, low-cost biological models, able to recapitulate in a dish a tissue-like environment, something that is essential in cardiac research. In the last years, the development of high-throughput platforms and softwares to do cardiac electrophysiological studies using zebrafish embryos/larvae has provided novel approaches to perform non-invasive cardiac assays aimed to do drug screening (Letamendia et al., 2012; Parker et al., 2014; Fuad et al., 2018; Gaur et al., 2018; Sharma et al., 2018).

## Electrophysiology of the Zebrafish

### Basal Electrocardiogram, Similar to That of Humans

Electrocardiogram has been the most extensively used routine technique for the evaluation of human cardiac diseases. Similarly, it has become a powerful tool for recording cardiac activity *in vivo*, as well as *ex vivo* in isolated hearts under Langendorff perfusion, in a number of laboratory animal models. These include medium-size fish like carp and tilapia (Yoshikawa et al., 1988). Milan et al. (2006) developed a method for recording *in vivo* the electrocardiogram of the small adult zebrafish. Unlike previous assays, limited to the visualization of the hearth rhythm in the transparent embryo (Milan et al., 2003), electrocardiography allowed for a detailed analysis of the cardiac depolarization, repolarization, and conduction, based on quantification of PR, QRS, or QT interval durations (Milan et al., 2006). Several research groups subsequently modified the recording technique in order to improve the feasibility, the signal-to-noise ratio, and the accuracy of the recordings (Liu et al., 2016; Lin et al., 2018; Skarsfeldt et al., 2018; Zhao et al., 2019). These studies demonstrated that, despite the evident anatomical differences between the human and the two chambered zebrafish heart, their electrocardiographic characteristics at baseline were highly comparable. The similarities included clearly recognizable P, QRS, and T waves, the QT interval duration, and slow heart rate, closer to that on humans. The zebrafish heart also receives autonomic innervation (Stoyek et al., 2016). Like in humans, but not in mice, the QT duration in the zebrafish is strongly dependent on the cardiac frequency (Milan et al., 2006) and must be corrected for variations in heart rate (QTc). Moreover, many of the ECG abnormalities observed in human hearts under pathological conditions can be reproduced in the zebrafish’s heart. Thus, in response to hyperkalemia, the electrocardiographic behavior of the zebrafish and the human hearts correlated: arrhythmia, AV block, widened QRS complex, and prominent peaked T wave. Similarly, they also showed ST segment depression and inverted T wave after myocardial infarction (Liu et al., 2016).

### Action Potential

The human cardiac AP consists of a rapid depolarization or upstroke followed by a rapid partial repolarization or phase 1, a plateau characteristic of the AP in large mammals, a final repolarization phase, and the recovering of the resting membrane potential. Although this basic shape appears in both chambers, important differences are observed between ventricular and atrial AP.

The most currently employed models for the study of cardiac AP are rodent, zebrafish, and, more recently, human cardiac myocytes derived from pluripotential stem cells (or hIPSC-CMs), which are becoming popular (Figure 1). However, direct electrophysiological recording in hIPSC-CMs requires a high degree of skill, so the alternative is to perform electrophysiological studies through voltage-sensitive dyes (Pfeiffer-Kaushik et al., 2018), despite providing relevant functional results, lacks direct measurements of voltage data.

APs from adult zebrafish ventricular myocytes were recorded for the first time by Brette et al. (2008) and were further characterized by Nemtsas et al. (2010). Those pioneer works showed that the resting membrane potential and the AP amplitude were similar in ventricular myocytes isolated from human and from zebrafish. Compared to human, the zebrafish AP lacked a visible spike or phase 1, but it showed a clear plateau. Since the resting heart rate is higher in zebrafish than in humans, the action potential duration (APD) is shorter. As in humans and in other larger mammals, APD shortens as the stimulation frequency increases because of a shorter duration of the plateau phase. Thus, the zebrafish ventricular AP resemble those of large mammals including human, in clear contrast to the triangular and almost rate independent APs recorded in mice and rats.

Another electrophysiological characteristic of the mammalian heart is the different shape and duration of atrial *vs* ventricular APs. This is also the case for zebrafish. Atrial AP amplitude is similar in mouse, human and zebrafish. However, the resting membrane potential is similar in zebrafish and human atrial myocytes, but different in mice, being less negative than in the atria of the latter.

The response of zebrafish heart to many pharmacological agents also resembles that of humans. Drugs that reduced human ventricular AP amplitude or shortened APD, such as TTX, lidocaine, nitrendipine, or nifedipine, had similar effects in the zebrafish ventricle (Nemtsas et al., 2010; Alday et al., 2014; Miranda et al., 2019). Some classical hERG channel blockers like E4031 or terfenadine prolonged AP both in the zebrafish and in humans (Nemtsas et al., 2010; Alday et al., 2014). However, the I_*Ks*_ or I_*to*_ blockers chromanol 293B, HMR 1556, and heteropodatoxin seemed to have no effect on zebrafish ventricular AP (Nemtsas et al., 2010; Alday et al., 2014; Ravens, 2018).

Besides, although the atrial APD is shorter in zebrafish than in human, both species share the shortening response to acetylcholine. This is an important characteristic of atrial but not ventricular cardiomyocytes, shared by both zebrafish and humans (Nemtsas et al., 2010).

Lastly, mutations and polymorphisms in cardiac ionic channels that prolonged APD in human ventricles also prolonged APD in zebrafish (Arnaout et al., 2007; Jou et al., 2013). Interestingly, knockdown of zebrafish orthologous genes related to atrial fibrillation (*Neurl* and C*and2*) in human heart had no effect on ventricular contractile function, but significantly prolonged atrial APD (Sinner et al., 2014).

### Cardiac Ion Channels in the Zebrafish

In the human cardiac AP, the initial upstroke is due to the opening of Na^+^ channels carrying the fast inward Na^+^ current, I_*Na*_. Phase 1 is due to the opening of the K^+^ channels responsible for the transient outward current, I_*to*_. The plateau phase results from the balance between the inward Ca^2+^ current, I_*Ca–L*_, and the outward rapidly activating delayed rectifier K^+^ current, I_*Kr*_. The final repolarization depends on the I_*Kr*_ and the inward rectifier K^+^ current, I_*K*__1_, and the resting membrane potential maintenance is mainly due to I_*K*__1_. Finally, the slowly activating delayed rectifier K^+^ current, I_*Ks*_, participates in the APD adaptation to adrenergic stimulation or increased heart rate.

The ionic currents that define the cardiac AP are conducted through specific ionic channels (Figure 1 and Table 1). In humans, the inward I_*Na*_ and I_*Ca–L*_ are carried through Nav1.5 and Cav1.2 channels, whereas the channel-forming proteins responsible for the outward potassium currents I_*to*_, I_*Kr*_, I_*Ks*_, and I_*K*__1_ are Kv4.3, Kv11.1 or hERG, Kv7.1, and Kir 2 (2.1, 2.2, 2.3), respectively. The zebrafish genome has orthologs of Nav1.5, Cav1.2, and the K^+^ channels Kv4.3, Kv7.1, and ERG (Rottbauer et al., 2001; Langheinrich et al., 2003; Novak et al., 2006; McDermott et al., 2007; Hassel et al., 2008; Abbas and Whitfield, 2009; Sanhueza et al., 2009; Lovering et al., 2018), whereas zebrafish Kir2 composition is dominated by an isoform hardly expressed in mammalian cardiomyocytes (Kir 2.4) (Hassinen, Minna, Jaakko Haverinen, Matt et al., 2015). However, as will be explained later, some cardiac ionic currents may be produced by different gene products in humans and zebrafish (Vornanen and Hassinen, 2016), and some channels might express in non-cardiac tissues but not in the heart (Buckingham and Ali, 2004; Coutts et al., 2006). The pacemaker current I_*h*_, the depolarizing Na^+^ and Ca^2+^ currents, and the repolarizing K^+^ current I_*Kr*_ have been recorded in the zebrafish heart (Warren et al., 2001; Brette et al., 2008; Nemtsas et al., 2010).

**TABLE 1:** Ionic currents responsible for the action potential in ventricular myocytes from adult human, zebrafish embryo, adult zebrafish, adult rat, and human-induced pluripotent stem cell-derived cardiac myocytes (hIPSC-CMs).

	Adult Human		Zebrafish Embryo		Adult Zebrafish		Adult Rat		hIPSC-CMs	
	Channel	Accesory subunit	Channel	Accesory subunit	Channel	Accesory subunit	Channel	Accesory subunit	Channel	Accesory subunit
I_*Na*_	Nav1.5	Navβ	Nav1.5	??	Nav1.5	Navβ	Nav1.5	Navβ	Nav1.5	??
I_*Ca–L*_	Cav1.2	β; α2δ	Cav1.2	β; α2δ	Cav1.2	β; α2δ	Cav1.2	β; α2δ	Cav1.2	??
I_*Ca–T*_	–	–	Cav3.2	–	Cav3.2	–	–	–	Cav3.2	–
I_*to*_	Kv4.3	MIRP1	–	–	–	–	Kv4.3; Kv4.2	MIRP1	Kv4.3	–
I_*Kr*_	hERG1		hERG?		hERG2		–		hERG1	
I_*Ks*_	Kv71.	MinK	–	–	Kv7.1	–	–	–	Kv7.1	–
I_*K*__1_	Kir2.1; 2.2; 2.3	–	Kir2.4	–	Kir2.4	–	Kir2.1; 2.2; 2.3	–	Kir2.1; ??;??	–
I_*K*_	–	–	–	–	–	–	Kv2.1	–	–	–

*Correlation with the different pore-forming α-subunits (Channel) and accessory proteins. Different font sizes represent different expression levels.*

#### Depolarizing Na^+^ and Ca^2+^ Currents

The sodium current was recorded by Warren et al. (2001) in both atrial and ventricular myocytes from adult zebrafish hearts. Although I_*Na*_ had similar amplitude in both chambers, atrial I_*Na*_ has a more negative inactivation half voltage and less voltage sensitivity than ventricular I_*Na*_. In the human heart, functional sodium channels that drive the I_*Na*_ consist of the pore-forming α-subunit Nav1.5 and different Navβ regulatory subunits, coded by the *hSCN5A* and the *hSCN1* to *4*β genes, respectively. Two orthologs of the Nav1.5 α-subunit, *zSCN5Laa* and *zSCN5Lab*, have been described in the zebrafish heart (Novak et al., 2006) and share 60% to 65% amino acid identity with *hSCN5A* (Novak et al., 2006; Chopra et al., 2010). Zebrafish also express single orthologs of *zSCN1* to *3*β, and two different *zSCN4* β genes, with homologies with human β genes between 50 and 54% (Chopra et al., 2007). Co-expression experiments on CHO cells showed that zβ-subunits modified the biophysical properties, including voltage dependence, of the sodium channel α-subunit zNav1.5 as in the heart of mammals. The atrial *vs* ventricular expression of α-subunits has not been explored, but the expression of β-subunits is clearly different, higher in ventricular than in atrial myocytes (Chopra et al., 2007). This difference in the expression of β-subunits could explain the biophysical differences between atrial and ventricular I_*Na*_.

As indicated above, the Ca^2+^ current was recorded for the first time by Brette et al. (2008), and later characterized by Nemtsas et al. (2010). Different stimulation protocols and pharmacological tools demonstrated that the current was actually a combination of the low threshold activated and Ni^2+^-sensitive I_*Ca–T*_, and the high threshold activated and nifedipine-sensitive I_*Ca–L*_. This is a striking difference between human and zebrafish adult hearts. In humans, I_*Ca–T*_, carried through Cav3.2 channels, contributes to the total calcium current during embryonic development and neonatal stage, but is absent from the adult heart (Vassort et al., 2006), where the plateau phase is maintained only by the I_*Ca–L*_, carried through the pore-forming Cav1.2 α-subunit plus the modulatory α2δ and β-subunits.

The *hCACNA1C* gene that encodes for the human Cav1.2 Ca^2+^ channel has two orthologs in zebrafish, named *zCANA1Ba* and *zCACNA1Bb* (Sanhueza et al., 2009), and the Cav1.2 is expressed at a similar degree in both zebrafish atrium and ventricle (Haverinen, Jaakko, Minna Hassinen, Surjya Narayan, Dash, and Matti Vornanen., 2018). Regarding the β-subunits, two *z*β*2* and two *z*β*4* genes have been found. The homology to the human genes in the SH3 and GK domains is 95–96% and the majority of amino acid substitutions are conservative (Ebert et al., 2008a,b; Chernyavskaya et al., 2012). The α2δ subunits are also present in zebrafish genome (Howe et al., 2013), sharing a 66% homology with human gene.

In humans, I_*Ca–T*_, carried through Cav3.2 channels, is present in the heart during embryonic development and neonatal stage, but is absent from the adult heart (Vassort et al., 2006). A robust I_*Ca–T*_ has been recorded in adult zebrafish atrial and ventricular myocytes (Brette et al., 2008; Nemtsas et al., 2010), and the pharmacological blockade of ICa-T strongly affects excitability in zebrafish embryonic heart (Alday et al., 2014).

The *zCACNA1G* gene, which encodes for the Cav3.1 channel, and the *zCACNA1Ha* (ortholog of the *hCACNA1H*), which encodes for the Cav3.2 channel, are present in the zebrafish genome (NCBI, 2020). In adult zebrafish, Cav3.1 is the most expressed cardiac calcium channel at the mRNA level, while Cav3.2 is expressed 100 to 1000 times less, suggesting that the current recorded in adult cardiomyocytes is transported by Cav3.1 (Haverinen, Jaakko, Minna Hassinen, Surjya Narayan, Dash, and Matti Vornanen., 2018). On the other hand, immunofluorescence staining revealed the presence of Cav3.2 channels in atrial and ventricular myocytes from zebrafish embryo (Alday et al., 2014).

#### Repolarizing K^+^ Currents

In both human and zebrafish, I_*Kr*_ is the main repolarizing current, although the I_*Kr*_ is generated by non-orthologous genes in human (*KCNH2* or *erg1*) and zebrafish (*KCNH6* or *erg2*) (Vornanen and Hassinen, 2016). Despite the fact that the similarity between both channels is very high, reaching 87% between the S1 segment and the CNBD (Cyclic Nucleotide Binding Domain), and exceeding 99% in the pore-forming domains (Langheinrich et al., 2003; Scholz et al., 2009), drug sensitivity is also comparable between both species.

When zERG was heterologously expressed in xenopus oocytes, the recorded current showed subtle differences from human hERG in its activation and deactivation kinetics as well as in voltage dependencies of activation and inactivation (Scholz et al., 2009). However, Scholz et al. demonstrated in an elegant experiment that, by applying a cardiac AP-shaped voltage pulse, that is, simulating physiological conditions, the currents generated by the zERG and hERG channels were almost identical (Scholz et al., 2009). For all the above evidence, the zebrafish heart is currently considered a good model for studying the ERG channel.

Although the K^+^ channel Kv4.3 gene *KCND3* has been found in zebrafish genome (Lovering et al., 2018), the corresponding cardiac I_*to*_ current has never been recorded (Nemtsas et al., 2010). This supports the idea that the channel may be present in non-cardiac tissues and not in the heart. In this sense, the A-type outward current, equivalent to cardiac I_*to*_, was recorded in skeletal but not in cardiac myocytes (Coutts et al., 2006).

Regarding Kv7.1 channels and I_*Ks*_ current, the results are controversial. Current recordings using patch-clamp technique, immunostaining of the channel, and APD inhibition experiments using specific blockers failed to find evidence of I_*Ks*_ in the adult and in the embryonic zebrafish hearts (Nemtsas et al., 2010; Alday et al., 2014). However, using a different technique to isolate cardiac myocytes, Abramochkin et al. (2018) recently showed that I_*Ks*_ is present in adult zebrafish ventricular myocytes. Human I_*Ks*_ is formed by the Kv7.1 channels together with the β-subunit MinK (Barhanin et al., 1996; Sanguinetti et al., 1996). The zebrafish heart expresses both the *zKCNQ1* and *zKCNE1* genes, encoding zKv7.1 and zMinK, respectively (Vernlund, 2011; Wu et al., 2014; Lovering et al., 2018). However, the low expression ratio of MinK in the zebrafish heart indicates that the current is mainly carried through Kv7.1 channels only. This absence of MinK in the channel complex can explain why the current kinetic is faster in the zebrafish than in the human heart (Abramochkin et al., 2018). In addition, it also explains why I_*Ks*_ blockers have no effect on the zebrafish AP, since the channel sensitivity to these compounds depends on the presence of this β-subunit (Busch et al., 1997; Lerche et al., 2000; Bett et al., 2006).

#### Background Channels

Genes responsible for the atrium-specific small conductance Ca^2+^-activated K^+^ SK channels, KCa2.1-3 (*KCNN1*, *KCNN2*, *KCNN3*); for the leak channels TASK1 and TASK3 (*KCNK3* and *KCNK9*); and the acetylcholine−activated current, I_*KACh*_, (*KCNJ3* and *KCNJ5*) have been reported in adult zebrafish heart. However, direct current recordings have shown that the acetylcholine-activated inwardly rectifying current, I_*KACh*_, is functional and significant in the zebrafish atrium, whereas TASK and SK channels are not relevant for the regulation of the atrial AP (Skarsfeldt et al., 2018).

## Zebrafish as a Model for Congenital and Acquired Long QT Syndrome (LQTS)

Long QT syndromes result in an excessive QTc interval duration (>450 ms in males and>460 ms in females), an indicator of high susceptibility to develop arrhythmia like torsade de pointes, ventricular fibrillation, and sudden death (Schwartz and Crotti, 2011). Congenital mutations that affect the functioning of cardiac ion channels lead to inherited LQTS, which affects around 1/5000 people worldwide. Although mutations in Na^+^, Ca^2+^, and different K^+^ channels can lead to LQTS, loss-of-function mutations in KCNQ1 or HERG are responsible for over 90% of the cases of inherited QT syndrome.

Besides, acquired LQTS can result from the use of drugs that block ionic channels but, in clinical practice, it is caused mainly by drugs that reduce the hERG-channel current (Sanguinetti and Tristani-Firouzi, 2006).

The zebrafish is an interesting animal model for the study of human LQTSs. In the zebrafish, mutations that reduced I_*Kr*_ current also induced prolongation in the QT interval. This is the case of the Zebrafish *breakdance* or *bre* mutant, the first mutation described in the zebrafish ERG (zERG), and the best characterized (Langheinrich et al., 2003). The *bre* zebrafish has a zERG-I59S mutation that reduced ERG protein trafficking to the membrane and, therefore, prolonged cardiac repolarization and QT interval duration in homozygosis (Meder et al., 2011; Liu et al., 2016). Two other mutations, I462R and M521K, also prolonged repolarization and led to LQTS in the zebrafish (Arnaout et al., 2007).

Regarding acquired LQTS, almost 100% of cases are caused by the blockade of the hERG channel by different and unrelated drugs (Sanguinetti and Tristani-Firouzi, 2006). It is important to highlight that the high sensitivity of hERG to be blocked by a wide variety of compounds is due to two specific amino acids, tyrosine at position 652 (Y652) and, especially, phenylalanine at position 656 (F656) (Mitcheson et al., 2000). Both amino acids locate in equivalent positions in the zERG ortholog (Y624 and F628) (Langheinrich et al., 2003). Therefore, classical hERG pharmacological blockers prolonged QT in the zebrafish, whereas non-prolonging drugs in humans showed no effect in the fish (Milan et al., 2006). Moreover, given the interest of the human potassium channel hERG as a source of acquired LQTS, most of the studies carried out on the zebrafish heart aimed to test the potential cardiotoxicity of novel drugs on cardiac hERG (Langheinrich et al., 2003; Mittelstadt et al., 2008; Shi et al., 2009).

## The Zebrafish Embryo and Larva for the Study of Cardiac Arrhythmia

### Cardiogenesis of the Zebrafish

Classically, teleosts are considered embryos until they hatch. Since hatching can occur over a wide period of time, ranging between 48 and 72 hpf in the case of zebrafish, it is difficult to define exactly the transition between embryo and larva (dechorionated embryo). Kimmel et al. (1995) established the end of embryonic period at 72 hpf, when the protruding mouth stage was attained. Cardiogenic differentiation for the future ventricle cells starts after 16 hpf, while in the future atrial cells, it occurs around 22 hpf, with the formation of a linear heart tube at 30 hpf (Bakkers, 2011). The heart begins to beat with no apparent direction at the onset of the pharyngula period (24–48 h), and at 26 hpf, the contraction occurs, which takes place in two parts, reflecting the development of the two chambers. At 36 hpf, the blood circulates, and the heart starts to bend, with the heart bending more prominent at 42 hpf. By this time, the heart beats with a rate of 180 bpm, preceding the atrial beat to the ventricle one. At the beginning of the hatching period (48–72 hpf), the heart tube continues bending to enhance the dorsal positioning of the atrium relative to the ventricle, and by then, the six pairs of aortic arches are present but not all are functional until the Pec-fin stage (60 hpf), when blood flows into all of them and through the subclavian loop (Kimmel et al., 1995).

It is well-known that vertebrate multichambered hearts required the coordination of their heart chambers to achieve the efficient blood flow throughout the organism. To achieve this, the cardiac conduction system (CCS) plays an essential role. Between 20 and 24 hpf, and prior to the beginning of the pharyngula period, when the heart starts its contractile activity, a linear conduction travels through the heart tube from the sinus venosus to the outflow tract, suggesting the presence of a SA nodal pacemaker activity. A significant delay in AV conduction is reported during chamber formation (36–48 hpf), and a rapid immature conduction network develops within the ventricle as the heart loops are made (72–96 hpf). Finally, at 21 dpf, a fully mature conduction pathway through ventricular trabeculae (equivalent of the His-Purkinje system), which allows fast apex-to-base activation pattern, is formed (Chi et al., 2008). Interestingly, through molecular identification using the marker islet-1, the triggering place of the pacemaker activity can be determined (Tessadori et al., 2012). Whereas in the adult zebrafish the primary pacemaking site occurs at the sinus venosus-atrial junction, propagating from there to the ventricle during atrial diastole (Tsutsui et al., 2010), in 3 dpf zebrafish, it is located in the right dorsal quadrant of the SA ring (Arrenberg et al., 2010).

### Cardiac Electrophysiology of the Zebrafish Embryo/Larva

The phylogenetic distance between fish and mammals is minimal in early developmental stages, when different vertebrate groups share general features, including cardiac function. For this reason, the use of zebrafish embryos/larvae, rather than adult specimens, provides a good model to perform cardiac physiology studies (Matrone et al., 2015).

In contrast to the adult zebrafish, due to the small size of the heart and the low signal-to-noise ratio in the early embryo/larva, electrocardiographic recordings are very uncommon. Forouhar et al. (2004) were able to record *in vivo* the ECG of zebrafish larvae at 5 days post fertilization (dpf) using micropipette electrodes, which differs from the existing methodology to study cardiac function in embryos/larvae, mainly based on optical mapping using voltage- and calcium-sensitive dyes, with limitations such as dye cytotoxicity, photobleaching, and low temporal resolution (Tamaddon et al., 2000). Five dpf larvae showed bimodal ECG, with atrial and ventricular depolarization waves (P and R waves, respectively) (Forouhar et al., 2004). Yu et al. (2010) established the dynamic changes of zebrafish larvae during developmental stages; thus, ECG revealed P waves and QRS complexes at 7 dpf, the T wave appeared at 14 dpf, and the three elements (P and T waves and QRS complex) adopted adult characteristics at 35 dpf. The effect of the antiarrhythmic drug amiodarone has been shown at the different stages as a prolongation of QRS interval, probably due to the blockade of the fast inward Na^+^ current (I_*Na*_), which causes a decrease of dV/dt (Yu et al., 2010). Later, Dhillon et al. were able to record all the components of the ECG (P, QRS, and T waves) in 48 hpf–5 dpf embryos/larvae using a microelectrode approach similar to that mentioned above (Dhillon et al., 2013).

In contrast to the difficulties to record ECGs, the transparency of the zebrafish embryos/larvae makes the direct observation of the heart beating pattern and the study of alterations in rhythm possible. That makes the zebrafish embryos/larvae a suitable model for the study of either congenital or acquired LQTS. In the zebrafish heart, atrium and ventricle beat coordinately with a 1:1 ratio. Screens for early developmental mutants identified mutations that led to severe arrhythmic phenotype, like the *hiphop* (hip) mutant embryo, whose beating pattern is 3:1 (the atrium contracts three times, but the ventricle once), or several mutants with fibrillating hearts (Chen et al., 1996). The previously mentioned *breakdance* (bre) mutant embryo has a beating pattern of 2:1 (Chen et al., 1996). Drugs that induced acquired LQTS in humans and in adult zebrafish caused bradycardia and 2:1 AV block in the embryo (Milan et al., 2003; Kopp et al., 2005). Knockdown of zERG function in the zebrafish embryo, as occurs in congenital LQT syndrome, caused similar effects (Langheinrich et al., 2003; Milan et al., 2003; Kopp et al., 2005; Arnaout et al., 2007). In humans, 2:1 functional AV block was reported in a newborn child with severe congenital LQTS, caused by homozygous mutations in hERG that led to absence of functional I_*Kr*_ (Hoorntje et al., 1999).

Although adult zebrafish have been used for the *in vivo* characterization of LQTS inducing mutations, unfortunately, most of these mutations, especially when expressed homozygously, create non-viable adult animals. Zebrafish embryos/larvae have the capacity of surviving without cardiovascular function up to 4 days (Hu et al., 2000), due to their ability to obtain oxygen by diffusion from the environment (Rottbauer et al., 2005). Thanks to this characteristic, it is noteworthy that the cardiac activity can be monitored in zebrafish embryos/larvae carrying lethal mutations up to late larval stages, while this causes *in utero* death in placental animals. Thus, as a part of a study of the LQTS mutations I462R and M521K of the zERG channel, Arnaout et al. recorded for the first time APs in atria and ventricle 48 hpf and demonstrated that the zebrafish embryo is a good model for the study of ERG dependent arrhythmia (Arnaout et al., 2007).

Another example of lethal mutations described in zebrafish is the *dead beat* (*ded*) (Stainier et al., 1996), which affects phospholipase Cγ1 gene (PLCγ1). A recent study has described the key role of PLCγ1 mutation in maintaining ventricular contractility in the zebrafish embryo. In *ded* mutant, the ventricular contractility declines over time, stopping by 60 hpf (Rottbauer et al., 2005). Further studies need to be done to understand the downstream cascade of PLCγ1 involved in contractility, although it seems to be mediated by the increase in Ca^2+^ stores by IP_3_. Finally, due to the fact that PLC functions downstream of FLT1 (gene of the vascular endothelial growth factor receptor 1—referred to as VEGFR1), and since the homology of *hFLT-1* and *zFLT-1* is high (∼51%), these findings could open a new insight into the treatment of HF exploring the VEGF–PLCγ1 pathway. It would suppose an alternative to the current treatments, aimed at improving cardiac contractility through changes in Ca^2+^ handling, which carry the risk of inducing arrythmias.

Regarding the cardiac AP, characteristics of the ventricular AP and the ionic channels responsible for the electrical activity in zebrafish embryos/larvae have been described at three different developmental stages: 48, 72, and 96 hpf (Alday et al., 2014). Thus, in zebrafish embryos/larvae, pharmacological inhibition demonstrated that the AP upstroke depends on both Na^+^- and T-type Ca^2+^ currents, and the plateau phase depends on L-type Ca^+2^ channels. The AP repolarization and diastolic potential depends on ERG K^+^ channels. Immunofluorescence staining confirmed the presence of the channels observed in adult Nav1.5, Cav1.2, Cav3.2, and ERG in the embryonic/larval heart. Another common feature of embryonic and adult APs is the absence of a spike and dome shape, mainly because of the absence of phase 1. Again, no effect was observed after pharmacological blockade and in immunostaining experiments, indicating that human I_*to*_ and I_*Ks*_ are absent from the heart of the embryonic/larval and adult zebrafish (Nemtsas et al., 2010; Alday et al., 2014). As mentioned above, I_*Ks*_ develops later and is only present in adult ventricle (Abramochkin et al., 2018).

Finally, in studies with the zebrafish mutant *slow mo* (*smo*), named for its slow heart rate, the T-type Ca^2+^ current, the rapid delayed rectifier K^+^ current, I_*Kr*_, and the hyperpolarization-activated pacemaker Na^+^ and K^+^ current, I_*h*_, have been recorded. The reduction of the I_*h*_ caused the low heart rate of the *slo mo* mutant (Baker et al., 1997).

## Zebrafish Embryos/Larvae for the Study of Cardiac Pathologies and Their Therapies

Whole animal experimental models are essential to understand the molecular mechanisms of diseases and are key to validate the therapeutic potential of new drug entities. Opposite to *in vitro* and *ex vivo* models, they offer a complete and integrated view of animal physiology, covering important issues such as drug absorption, distribution, metabolism, and excretion (ADME), which are missed in cell culture. Zebrafish has emerged as an alternative to conventional *in vivo* mammal models, which are more expensive and time-consuming, and therefore are restricted to late stages of drug screening (Delgado et al., 2004). The fact that zebrafish embryos/larvae can be maintained at high densities, together with the straightforward procedure for drug administration, make them suitable for high-throughput assays, enhancing the efficiency of several steps during drug development process (Zon and Peterson, 2005).

Since zebrafish has morphological and molecular bases of tissues and organs similar to humans, including heart, it could provide a good model to study cardiac pathologies and drug responses. Zhu et al. (2014) used the zebrafish embryo (48 hpf) treated with verapamil as a HF model to evaluate potential therapeutic agents. They found that a 30-min treatment with 200 μM of verapamil is the optimal condition for inducing changes in the cardiovascular system, compatible with those observed in human HF (pericardial edema, venous blood congestion, reduced cardiac output, and slowed blood flow) (Zhu et al., 2014). Eight therapeutic drugs for HF treatment, approved either by the USA Federal Drug Administration (FDA) (LCZ696, digoxin, irbesartan, metoprolol, enalalpril, and hydrochlorothiazide) or by China’s State Food and Drug Administration (CFDA) (qiliqiangxin capsule and shenmai injection), were tested. The eight drugs significantly reduced HF in the zebrafish embryo after 4.5 h of treatment, demonstrating the suitability of this model to screen HF drugs, saving time, and reducing costs and drug attrition at later stages of drug development (Zhu et al., 2018).

Zou et al. established a novel hypoxia/reoxygenation (H/R) model in zebrafish larvae to simulate myocardial ischemia/reperfusion injury (MIRI) as an alternative for *in vivo* screening of MIRI therapeutics. The optimal conditions to simulate MIRI consisted of 48 h of hypoxia followed by 2–5 h of reoxygenation. Besides heart dysfunction, they found upregulation of myocardial injury markers such as cardiac troponin (TNNT2), ventricular natriuretic peptide, and hypoxia-inducible factor 1-alpha (HIF1α), and increased red blood cells (Zou et al., 2019).

## Adult Zebrafish and Zebrafish Embryos/Larvae in Industry

Traditionally, two types of screening tests have been used during the drug discovery process by the pharmaceutical industry. The first one, a purely *in vitro* approach, consists on identifying potential drug candidates based on their ability to bind molecular targets. The second one is a phenotypic screening with the objective of identifying drugs capable of modifying a disease phenotype, following *in vitro* or *in vivo* methodologies (cells, tissues, or whole organisms) (Swinney and Anthony, 2011). The phenotypic screening is a very reliable strategy and used to be the mainstay of drug development, with the inconvenience of slowing down the pipeline due to the difficulties to find out what targets are being achieved. For this reason, the pharmaceutical industry focused its efforts on target-based approaches, which are faster even though they have a lower success rate. Zebrafish belongs to this second group (phenotypic assays). The innovation reached within *in silico* and *in vitro* target identification has brought phenotypic assays back in trend (Kotz, 2012).

Zebrafish offers an *in vivo* model to perform comprehensive toxicological and long-term efficacy tests, as well as safety studies before moving to the clinical phase, filling the gap found between *in vitro* and *in vivo* tests reported by the pharmaceutical industry (Parker et al., 2014). Besides, zebrafish embryos/larvae have an extra advantage compared to adult specimens: the employment of larvae up to 5 dpf represents a replacement alternative in animal research (Dhillon et al., 2013) and, therefore, has a direct impact in the Replacement, Reduction and Refinement (3Rs) strategy, an essential aspect to fulfill the ethical standards required in the pharmaceutical and chemical industries (Avey et al., 2015).

### Zebrafish for Studying Drug Toxicity

Probably, the most widespread use of the zebrafish embryo/larva in industry is the evaluation of potential toxicity of chemicals and drugs. The small size of the embryo/larva provides a system where only a minimal amount of water-soluble chemicals is required and can be directly added to the embryos/larvae (Aspatwar et al., 2019). The compounds with poor water solubility, however, need to be injected into the fish to ensure enough exposure. The negative consequence of this feature is the reduction of the chemical pool to be tested and, therefore, the experimental throughput (Cassar et al., 2020). The main advantage of the zebrafish embryo/larva lies in the possibility to offer an economic and quick assessment in a whole vertebrate organism covering the entire organogenesis period, supposing an alternative to commonly used *ex vivo* models such as whole embryo culture or embryonic stem cells (Cassar et al., 2020).

Dhillon et al. optimized the recording of ECG in zebrafish larvae to perform reliable cardiotoxicity assays. The assay was sensitive and specific to detect drug-induced changes in QT intervals, and the results were reliable, reproducible, and compatible with those obtained from adult zebrafish (widening of QRS complex and T wave). The drugs terfenadine and haloperidol prolonged the QT interval and caused 2:1 AV block with similar features to those observed in humans. Therefore, the assessment of ECG in zebrafish embryos/larvae could be a good surrogate for cardiac dysfunction in humans. Since zERG seems to be more sensitive to QT prolonging drugs than hERG, the aforesaid approach could be a good alternative to test the cardiotoxicity of drugs in development (Dhillon et al., 2013).

#### Ecotoxicity Studies

One area of research with zebrafish is the study of the adverse effects of xenobiotics on animal growth and development. For decades, zebrafish embryos/larvae have been used as biosensors in ecotoxicity studies to test the presence of contaminants and to understand how environmental endocrine disruptors, contaminants and drugs affect human development and health (Vliegenthart et al., 2014; Aluru, 2017; Aedo et al., 2019; Silveira et al., 2019). The model is useful to study the cardiac toxicity induced by pollutants from embryonic to adult stages, showing cardiac malformations and changes in heart rate and in ejection fraction (Wang et al., 2020). These environmental toxicity assays are often performed with zebrafish embryos in combination with other standard models such as *Daphnia magna* and algae (Clements et al., 2012), while adult fish are kept for evaluating behavioral toxicity (Chagas et al., 2019).

#### Evaluation of Teratogenic Potential of Drugs

During the drug development process, prior to the marketing of drugs, it is also necessary to evaluate teratogenic potential as part of the safety studies. The employment of zebrafish embryos/larvae has the advantage of providing both drug cardiovascular toxicity and cardiovascular development effects (Chang et al., 2014; Caballero and Candiracci, 2018). Teratogenic studies are done in embryos/larvae from 5 to 96 hpf, covering from late blastula/early gastrula stages to fully developed organs (Beekhuijzen et al., 2015). The most remarkable parameters obtained from hemodynamic evaluation are beating rate, cardiac output, fractional area change, and shortening and blood flow velocity (Shin et al., 2010). Functional analysis includes evaluation of heart pumping efficiency, while structural analysis provides information about the heart size. These studies require rapid imaging techniques, as well as robust software to process the information (Zakaria et al., 2018).

#### Cardiotoxicity Studies

Several laboratories offer, in addition to global safety studies (Cornet et al., 2017), specific studies to test the potential toxicity on particular organs like the heart (Alzualde et al., 2018). For cardiovascular drug toxicity, the zebrafish embryos/larvae allow a more reliable prediction of cardiotoxicity than cellular systems and yield similar predictive performance to previous validation meta-studies performed with dogs, the standard preclinical model for predicting cardiotoxic liabilities prior to clinical phases (Dyballa et al., 2019).

Since cardiovascular physiology is conserved in humans and zebrafish, the effect of many drugs is comparable between both species (MacRae and Fishman, 2002). Zebrafish embryo has been used to assess the impact of cigarette smoke, alcohol, and recreational drugs on the cardiovascular system in order to understand the effects of maternal consumption during pregnancy in the fetal development, showing alterations in heart rate, morphology, development, and function (Ellis et al., 2014; Mersereau et al., 2015; Li et al., 2016).

Milan et al. performed a cardiotoxicity study using a library of 100 compounds, 23 of them well-known QT prolonging drugs in human. Their results showed that only 18 out of the 23 drugs tested induced bradycardia and AV block in zebrafish, and the other 5 were false-negatives because the drug was not properly absorbed. Antisense KCNH2 oligonucleotide injection yields bradycardia in zebrafish, demonstrating the correlation between I_*Kr*_ blockade and bradycardia.

Cardiotoxicity assays are performed integrating fluorescent reporter genes into the genome of the embryos/larvae. The resulting zebrafish express a green fluorescent protein (GFP) in the heart, which makes the direct observation of the main cardiac processes possible (fluorescent heart images can be seen in Alday et al., 2014; Bloomekatz et al., 2017). GFP transgenic embryos/larvae are incubated with the molecules of interest and few-second videos of the beating heart are recorded to analyze cardiac physiological functions that may be indicative of toxicity (Hoage et al., 2012). In order to analyze as exhaustively as possible potential cardiac dysfunctions, some high-throughput screening platforms have been developed to analyze and quantify the impact of drugs and diseases in cardiac and vascular performance. These platforms usually combine microscopic high-definition videos taken *in vivo* with software analyses through advanced algorithms that allow the quantification of multiple relevant cardiovascular parameters like heart rate, arrhythmia, AV blockage, ejection fraction, or blood flow (De Luca et al., 2014; Fuad et al., 2018; Dyballa et al., 2019).

### The Zebrafish as a Tool to Identify Novel Therapies for Human Cardiovascular Disease

Drug efficacy studies are a cornerstone in the development of new drugs. Pharmaceutical companies are the first to be interested in knowing the effectiveness of new compounds before reaching clinical development, where the increase in costs is dramatically significant. Since the zebrafish embryo/larva has the ability to oxygenate through diffusion, mutations and diseases that cause severe cardiac phenotypes, including non-contracting hearts, can be analyzed (Asnani and Peterson, 2014).

On the other hand, one therapeutic area of interest to the medical community and pharmaceutical industry is the cardioprotection of patients at cardiovascular risk. In these studies, zebrafish embryos with green fluorescent heart are dechorionated and incubated with the cardiotoxic compound doxorubicin in combination with the potentially protective molecules of interest. At the endpoint, larval heart and vessels are imaged and analyzed.

Finally, the regenerative capacity of the zebrafish’s heart gives the opportunity to study therapies for heart injury and scar formation after myocardial infarction. One interesting research program aimed at generating new cardiac therapeutic strategies in the field of cardiac regeneration is REANIMA^1^, a Europe-wide project coordinated by the Spanish CNIC where zebrafish play an essential role due to their capacity to regenerate the heart. In adult mammals, including humans, the regenerative capacity of the heart is residual and insufficient to recover its function naturally. This EU-funded project aims at studying, analyzing, and thoroughly describing the regeneration mechanisms present in zebrafish to transfer them to humans, thus addressing translating knowledge of regenerative biology from the laboratory to clinical applications, in this case regeneration of the heart.

#### Platform Development to Perform Cardiac Assays in Zebrafish Embryos/Larvae

The demand of automated and high-throughput platforms to allow cardiac electrophysiology studies using zebrafish embryos/larvae has driven the efforts of research centers and biotechnological industries to develop experimental protocols to accurately assess cardiac function in this singular model. The assessment of cardiac endpoints in zebrafish embryos/larvae based on video recordings allows non-invasive, long-term *in vivo* studies. These emerging approaches suppose a good alternative for preclinical drug-discovery and toxicology studies. In the last years, several groups have concentrated their efforts in the development of different technologies of great utility for the pharmaceutical industry.

Different platforms and software, such as ZebraPace (Zebrafish Precise Algorithm for Cardiac-rhythm Estimation), MicroZebraLab^TM^ (v3.5, ViewPoint, Lyon, France) or DanioVison system (Noldus Information Technology, Netherlands), have been developed to quantify the contractility, heart rate, incidence of arrhythmia, and blood flow in zebrafish embryos/larvae (Parker et al., 2014; Gaur et al., 2018; Kang et al., 2018; Wang et al., 2020).

The employment of transgenic zebrafish expressing GFP within cardiac and/or vascular cells allows to do high-throughput cardiotoxic assays in 5–72 hpf zebrafish embryo for the quantification of heart rate, blood flow, and pericardial area, among other parameters (Yozzo et al., 2013).

Most of the image-based heart rate assays are limited to the analysis of one embryo per image field, but the challenge of performing this assay from multiple embryos per field was recently achieved. They have been designed different platforms able to record and process videos in plates with multiple embryos (2 dpf) using either bright-field or fluorescence imaging, circumventing the need of immobilizing the specimen either by anesthesia or dynamic force (Letamendia et al., 2012; Martin et al., 2019). In both cases, the reliability of the results has been compared with well-established methodologies.

To tackle the limitation due to manual manipulation of zebrafish specimens and the need of agarose-embedding, different groups have developed platforms integrating micro-echocardiography, microfluidic chips, high-resolution imaging system, and several devices for robotization. The strength of these platforms relies on its automation and high throughput (10–20 embryos/larvae simultaneously) (Letamendia et al., 2012; Fuad et al., 2018).

## Summary and Future Direction: Humanization of Zebrafish

The main limitation of animal models, even those with high homology with humans, occurs in the moment of translating the results to human biology due to the well-known inter-species variability. Human-induced pluripotent stem cells seem to be the closer *in vitro* approach to human physiology; nevertheless, the difficulties to recapitulate *in vitro* the conditions of a tissue-like environment is the Achilles heel of this model, when cultured in 2D. The recreation of organ-like environment is especially necessary in cardiac studies, where the dynamic factors (motion and stretch), the electrical communication, and the paracrine signals are essential for proper cardiac function (Giacomelli et al., 2017). The humanization of zebrafish can be achieved by substituting an endogenous gene with its human ortholog, contributing to obtain solid evidences regarding drug–target interactions (Cornet et al., 2018). This approach is especially relevant because the percentage of human disease-related genes with functional zebrafish orthologs is around 83% (Howe et al., 2013) and, therefore, most of human pathologies can be faithfully recapitulated in zebrafish. This issue, together with the fact that the main organ implicated in drug metabolism (liver) is functional from early development (Parng, 2005) and that the zebrafish physiology is able to recapitulate mammalian drug metabolism characteristics (ADME), makes the zebrafish humanized embryos/larvae one of the most convenient strategies for pharmaceutical industries. Cornet et al. suggest the use of CRISPR/Cas9 to facilitate this procedure, which could provide new insights to perform preclinical target validation, with the advantage of providing a dual view due to the possibility of evaluating drug toxicity and drug efficacy simultaneously (Cornet et al., 2018).

## Author Contributions

OC and MG conceptualized, designed, and wrote the manuscript. LE and MH-V designed and wrote the manuscript. All authors edited and proofread the manuscript and approved the final version.

## Conflict of Interest

The authors declare that the research was conducted in the absence of any commercial or financial relationships that could be construed as a potential conflict of interest.

## Footnotes

1 https://cordis.europa.eu/project/id/874764
